# Case Report: Partial Response Following Nivolumab Plus Docetaxel in a Patient With *EGFR* Exon 20 Deletion/Insertion (p.N771delinsGF) Mutant Lung Adenocarcinoma Transdifferentiated From Squamous Cell Carcinoma

**DOI:** 10.3389/fcell.2021.755135

**Published:** 2022-01-10

**Authors:** Lingling Zhu, Yanyang Liu, Honglin Gao, Jiewei Liu, Qinghua Zhou, Feng Luo

**Affiliations:** ^1^ Lung Cancer Center, West China School of Medicine, West China Hospital of Sichuan University, Chengdu, China; ^2^ Key Laboratory of Drug Targeting and Drug Delivery System of the Education Ministry, Sichuan Engineering Laboratory for Plant-Sourced Drug, Sichuan Research Center for Drug Precision Industrial Technology, College of Polymer Science and Engineering, West China School of Pharmacy, Sichuan University, Chengdu, China

**Keywords:** non-small cell lung cancer, immune checkpoint blockade, EGFR exon 20 mutation, histological transformation, immune therapy

## Abstract

The histological transformation from lung squamous cell carcinoma (LUSC) to lung adenocarcinoma (LUAD) and p. N771delinsGF mutations in *EGFR* exon 20 (ex20) are exceedingly rare in non–small cell lung carcinoma (NSCLC). *EGFR* ex20 mutations are insensitive to EGFR tyrosine kinase inhibitors in NSCLC. Here, we present a 76-year-old male smoker harboring LUAD with a novel p. N771delinsGF deletion/insertion mutation in *EGFR* ex20 transdifferentiating from advanced LUSC after chemoradiotherapy. The patient presented reduced hydrothorax and relieved tightness with the treatment of nivolumab plus docetaxel and carboplatin after the failure of second-line chemotherapy. The case highlights the importance of rebiopsy and molecular retesting after the progression of lung cancer and supports the idea that the combination of immune checkpoint blockade and chemotherapy may be an attractive option for patients with *EGFR* ex20 mutations associated with LUSC–LUAD transformation.

## Introduction

Lung cancer has the highest lethality rate among all cancers. Non–small cell lung carcinoma (NSCLC) is a major subtype of lung cancer, accounting for 85% of lung cancer cases, including lung adenocarcinoma (LUAD), lung squamous cell carcinoma (LUSC), and large cell carcinoma histologic subtypes. LUAD and LUSC harbor different features (Krause et al., 2020; Wang et al., 2020). Accumulating evidence from animal experiments and clinical observations indicates phenotypic plasticity in lung cancer cells, such as transdifferentiation of LUAD to LUSC or to small cell lung cancer. This phenotypic transition is a novel cellular mechanism for drug resistance in chemotherapy and in *EGFR*-targeted therapy (Hou et al., 2017). However, information regarding the transformation from LUSC to LUAD remains limited.

Studies have shown that approximately 12% of patients with *EGFR*-mutant NSCLC harbor insertion mutations in exon 20 (ex20ins), which are insensitive to first- and second-generation EGFR tyrosine kinase inhibitors (TKIs) (Riess et al., 2018). LUAD and LUSC of adenosquamous carcinomas (ASCs) also share a monoclonal origin, with *EGFR* mutations being the most common oncogenic driver in the Asian population (Lin et al., 2020). However, no information is available regarding the relationship between *EGFR* ex20ins mutations and the molecular mechanisms of driving LUSC transdifferentiating to LUAD.

Here, we describe a case of a patient with LUAD harboring a novel p. N771delinsGF deletion/insertion (delins) mutation in *EGFR* ex20 who experienced transdifferentiation from LUSC after failure of chemotherapy and subsequently responded favorably to immune checkpoint blockade (ICB) plus chemotherapy.

## Case Presentation

**FIGURE 1:**
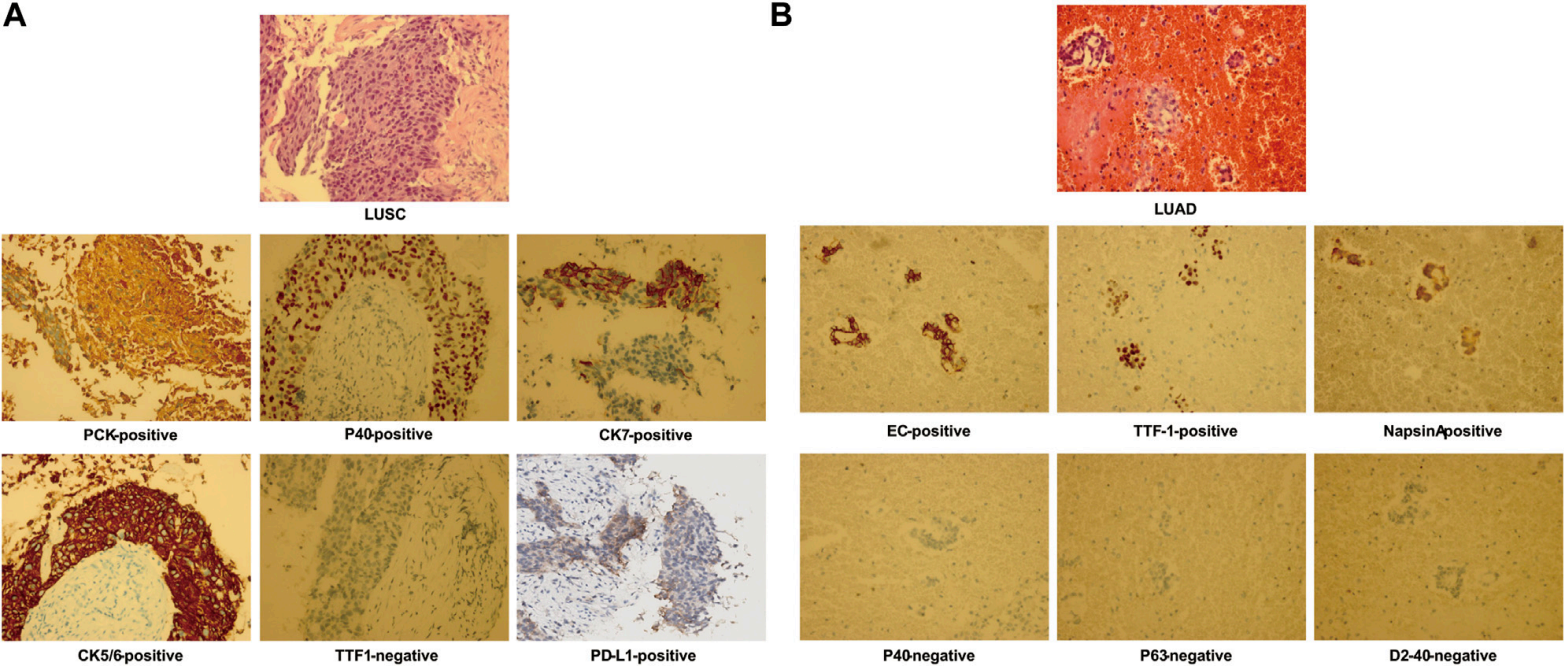
Hematoxylin–eosin staining and immunohistochemistry staining from pulmonary biopsy shows lung squamous cell carcinoma (LUSC) **(A)** and of pleural fluid–exfoliated cells shows lung adenocarcinoma (LUAD) **(B)**.

**FIGURE 2:**
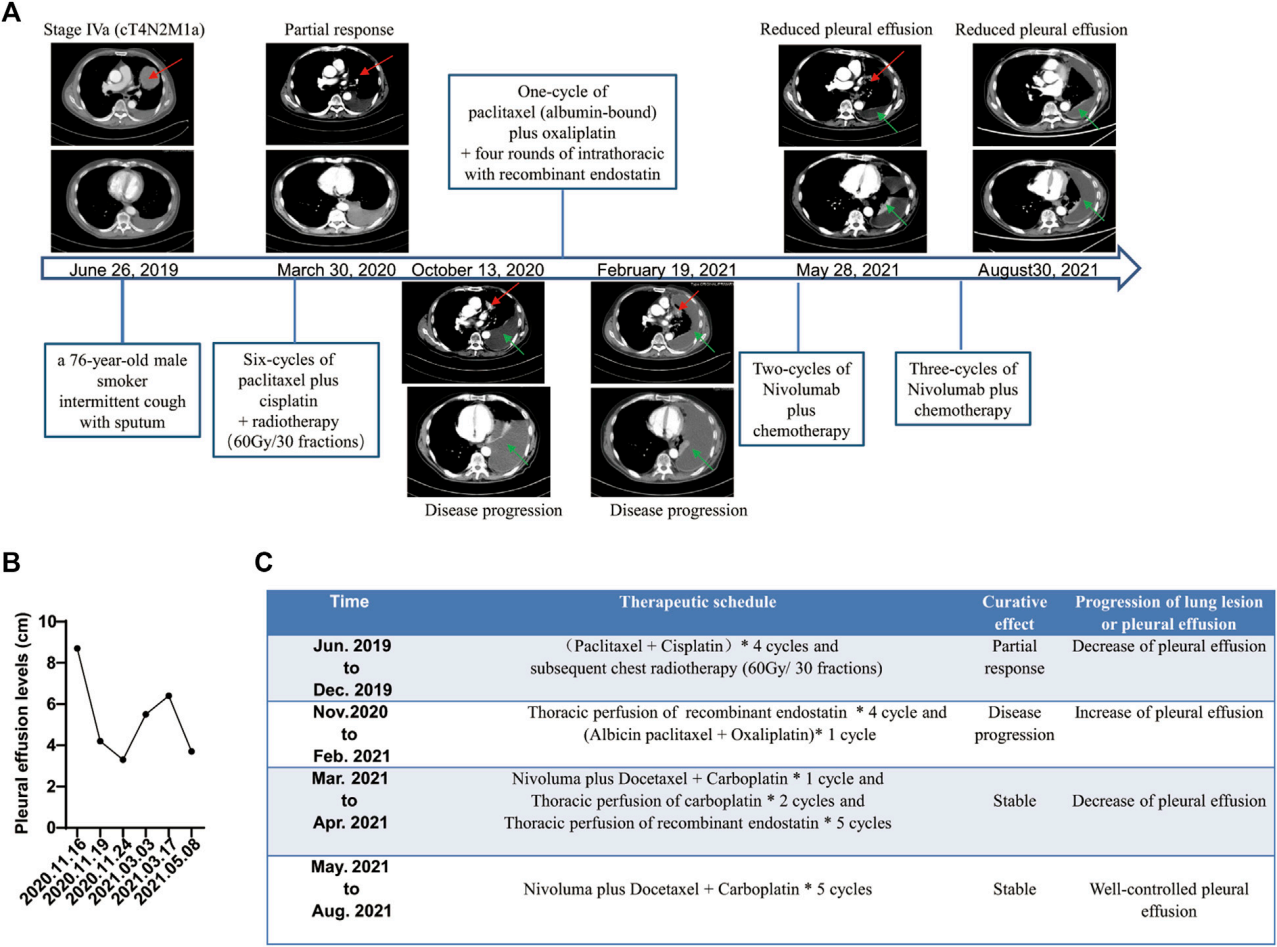
Summary of treatment schedule and associated therapeutic effects. **(A)** Computed tomography (CT) from June 26, 2019, shows a lesion in the left lung and enlarged left mediastinal and hilar lymph nodes (red arrows). CT from March 30, 2020, shows partial response after six cycles of paclitaxel plus cisplatin chemotherapy and subsequent radiotherapy (red arrows). CT from October 13, 2020 shows progressive spread of the tumor within the chest associated with left pleural effusion (green arrows). CT from February 19, 2021, shows increased pleural effusion after one cycle of second-line chemotherapy (green arrows). CT from May 28, 2021, to August 30, 2021, shows well-controlled pleural effusion after five courses of nivolumab plus chemotherapy and four rounds of intrathoracic chemotherapy with recombinant endostatin followed by two rounds of that with recombinant endostatin plus carboplatin (green arrows). **(B)** Pleural effusion levels before and after treatment with nivolumab plus chemotherapy by B-mode ultrasound. **(C)** Treatment course and results.

In June 2019, a 76-year-old male smoker weighing 50 kg with no notable medical, family, or psychosocial history was admitted to the hospital with a complaint of intermittent cough with sputum for >1 month. Chest computed tomography (CT) revealed a left upper lobe lung mass with multiple metastases involving the left pleura, left pleural effusion, and left mediastinal and hilar lymph node enlargement. Lung fine-needle aspiration biopsy confirmed the diagnosis of low-differentiated LUSC at stage IVA (cT4N2M1a) (Figures 1A, 2A). The immunohistochemistry of pulmonary biopsy samples was positive for PCK, P40, CK7, CK5/6, and PD-L1 (40%) and was negative for TTF1. Genetic abnormalities such as amplification of *CCDN1*, *CCDN2*, *FGF19*, *FGF3*, *FGF4*, *FGFR1*, *KRAS*, and *PIK3CA*; ex2 missense mutation of *CDKN2A*; and ex5 code-shift mutation of *TP*53 were observed in the primary lung cancer lesion by using next-generation sequencing (NGS; 520-gene panel, Oncoscreen plus, Burning Rock Biotech, Guangzhou, China). Plasma NGS demonstrated amplification of *CCDN1*, *FGF19*, *FGF3*, and *FGF4*; ex2 missense mutation of *CDKN2A*; ex5 shift mutation of *TP53*; and 11.1 mutations/Mb (Table 1).

**TABLE 1:** Next-generation sequencing results of pulmonary biopsy and plasma before treatments and of pleural fluid–exfoliated cells after chemoradiotherapy.

Mutant genes	Primary lung cancer before treatment (lung squamous cell carcinoma)			Plasma before treatment (lung squamous cell carcinoma)			Pleural effusion ctDNA after chemo-radiotherapy (lung adenocarcinoma)		
	Mutation type	Mutation location	Mutation abundance	Mutation type	Mutation location	Mutation abundance	Mutation type	Mutation location	Mutation abundance
CCDN1	Amplification	11q13.3	CN: 8.9	Amplification	11q13.3	CN: 3.1	—	—	—
CCDN2	Amplification	12p13.32	CN: 3.6	—	—	—	—	—	—
FGF19	Amplification	11q13.3	CN: 8.3	Amplification	11q13.3	CN: 3.3	—	—	—
FGF3	Amplification	11q13.3	CN: 7.7	Amplification	11q13.3	CN: 3.2	—	—	—
FGF4	Amplification	11q13.3	CN: 7.6	Amplification	11q13.3	CN: 2.9	—	—	—
FGFR1	Amplification	8p11.22	CN: 4.9	—	—	—	—	—	—
KRAS	Amplification	12p12.1	CN: 4.0	—	—	—	—	—	—
PIK3CA	Amplification	3q26.32	CN: 3.9	—	—	—	—	—	—
CDKN2A	Exon 2 missense mutation	c.242c>T p.Pro81Leu	65.05%	Exon 2 missense mutation	c.242c>T p.Pro81Leu	6.14%	—	—	—
TP53	Exon 5 code-shift mutation	c.545_552del p.Cys182fs	53.07%	Exon 5 code-shift mutation	c.545_552del p.Cys182fs	7.19%	—	—	—
TMB	—	—	—	—	—	11.1 Muts/Mb	—	—	6.72 Muts/Mb
EGFR	—	—	—	—	—	—	p.N771delinsGF deletion insertion mutation	Exon 20	0.5%
AKT1	—	—	—	—	—	—	p.E17k	Exon 3	0.8%

From July to December 2019, the patient was treated with six cycles of paclitaxel plus cisplatin chemotherapy and subsequent radiotherapy (60 Gy/30 fractions at 2 Gy per day). He experienced an initial partial response with a decrease in tumor size (from 8.5 × 9.6 × 10.4 cm to unmeasurable). However, he was readmitted to our hospital with breathlessness and tightness of the chest. Chest CT in October 2020 showed that the amount of pleural effusion in his left chest had notably increased. LUAD cells (EC-positive, TTF-1–positive, napsin A–positive, P40-negative, P63-negative, D2-40–negative) were found by cytological examination of pleural effusion (Figure 1B). Unfortunately, the patient showed poor compliance resulting from the side effects of kidney function and gastrointestinal symptoms of cisplatin during first-line chemotherapy. We have demonstrated the safety and efficacy of the combination of paclitaxel plus oxaliplatin in pretreated advanced NSCLC (Xie et al., 2004); oxaliplatin was administered in the second-line treatment instead of cisplatin.

However, after one cycle of second-line treatment (albumin-bound paclitaxel plus oxaliplatin) and four rounds of recombinant endostatin (an antiangiogenesis agent), the disease continued to progress. Chest CT and B-mode ultrasound revealed an increase in the amount of pleural effusion (from 4.2 cm in November 2020 to 6.4 cm in February 2021; Figures 2A, B). A novel p. N771delinsGF indel mutation in *EGFR* ex20, a p. E17k mutation in *AKT1* ex3 and 6.72 mutations/Mb were identified in the pleural fluid ctDNA by a hybrid-capture NGS method using a 1,021-gene panel (Table 1).

Five cycles of nivolumab plus docetaxel and carboplatin therapy as third-line therapy were initiated on March 19, 2021. The amount of pleural effusion then decreased (from 6.4 cm in March to 3.7 cm in May by B-mode ultrasound, Figure 2B) with well-controlled symptoms of breathlessness and chest tightness, and the lung lesion appeared stable. Moreover, an improved Eastern Cooperative Oncology Group performance status score (from 2 to 0) was observed in the patient after treatment with nivolumab plus chemotherapy. Therapeutic response was stable until the last follow-up on August 31, 2021, according to the well-controlled and unmeasurable lung lesion, decreased pleural effusion tested by chest CT, and well-controlled symptoms (Figure 2A). During the whole process of treatment with immunochemotherapy, the patient experienced no severe side effects.

## Discussion

We report a patient who was initially diagnosed with LUSC and was then found to have LUAD cells in the pleural effusion after chemotherapy resistance. One explanation for this is that the LUAD cells may have arisen from an original LUAD component of ASCs in our case. However, the patient had no shared mutations between the two genomic profiles of LUAD and LUSC detected by NGS. This is not in line with previous studies suggesting that LUAD and LUSC of ASCs share a monoclonal origin (Lin et al., 2020). Such a case may present more like two different pathological types. Recently, emerging data have provided convincing evidence supporting a phenotypic transition in lung cancer, such as adenocarcinoma–to–squamous cell transdifferentiation (AST) (Hou et al., 2017). Animal studies suggest that the mechanisms of AST are associated with *Kras* activation and *Lkb1* deletion. Moreover, AST is observed during the development of chemotherapy resistance, suggesting that the transdifferentiation of lung cancer represents a novel mechanism of drug resistance (Han et al., 2014). Thus, we suggest that there may have been a transdifferentiation from LUSC to LUAD which led to chemotherapy resistance in our case. Although this hypothesis needs to be further confirmed, the high plasticity of lung cancer indicates this possibility.

Notably, LUSC and LUAD in our report were diagnosed using bronchoscopy biopsy specimens and cytological examination of exfoliated cells in pleural effusion, respectively. This highlights the importance of repetitive and comprehensive sampling and molecular testing, including that of tissues (biopsy specimens or surgery), plasma, and body fluid, both before and after treatments. The overall genomic profiles of both LUAD and LUSC in our case were detected by NGS, which allows sequencing of a high number of nucleotides (Mosele et al., 2020) and facilitated identification of the novel *EGFR* ex20 p. N771delinsGF mutation in our case. However, it has been reported that each fluid type (blood, urine, or saliva) still retains its own characteristic signature based on its unique molecules (El-Mogy et al., 2018). For instance, cell-free DNA from body fluid supernatants has a higher detection rate and sensitivity for tumor-specific mutations than cell-free DNA from sedimented tumor cells and plasma from body fluid (Guo et al., 2019; Zhang et al., 2019). LUAD was detected by pleural effusion in our case, indicating that more information may be revealed from a pleural effusion sample compared with LUSC diagnosed by biopsy specimens and plasma before treatment.

LUAD with a novel p. N771delinsGF mutation occurring in *EGFR* ex20 was found in our case. The *EGFR* ex20ins mutations are diverse, accounting for approximately 12% of patients with EGFR mutations. The V769_D770insASV is the most common in *EGFR* ex20ins mutation (23.0%) (Tsiambas et al., 2016; Riess et al., 2018). Similar to other *EGFR* mutation subtypes, *EGFR* ex20in functions as an oncogenic driver. However, compared with those with *EGFR* ex19 deletion or ex21 L858R mutation, NSCLC patients with *EGFR* ex20ins mutations have reduced affinity for EGFR-TKIs. They experienced a shorter median survival than patients with EGFR-TKI–sensitive mutations (16.5 vs. 33.0 months) during EGFR-TKI therapy (Naidoo et al., 2015; Yang et al., 2020). Nowadays, targeted therapeutics such as amivantamab, mobocertinib, and poziotinib have shown initial efficacy in NSCLC patients with *EGFR* ex20ins (Köhler and Jänne, 2021); however, no targeted therapeutics have been approved for *EGFR* ex20ins-driven NSCLC by the US Food and Drug Administration. Currently, chemotherapy remains the suitable treatment for patients harboring these mutations in China (Naidoo et al., 2015). One study showed that metastatic NSCLC patients with *EGFR* ex20ins treated with platinum chemotherapy presented a more favorable overall survival (OS; median 20 vs. 12 months) compared with NSCLC without targetable alterations (Choudhury et al., 2021). In a single-institution retrospective study (n = 29) of NSCLC with *EGFR* ex20ins, the median progression-free survival (PFS) with platinum-based chemotherapy was 7.1 months (95% confidence interval [CI], 6.3–13.7), and the median OS was 3.2 years (95% CI, 1.92–not reached) (Shah et al., 2021). These data suggest that LUAD patients with EGFR 20ins mutation may respond to chemotherapy. However, the present patient’s disease remained unresponsive to second-line chemotherapy agents alone in our case, indicating an urgent need for the development of new therapeutic strategies.

Immunotherapy, such as ICB, has changed the treatment landscape in metastatic and recurrent NSCLC. Chemotherapy plus immunotherapy has shown particularly promising efficacy in advanced NSCLC patients (Leonetti et al., 2019). Although compared with *EGFR* wide-type LUAD, cases of NSCLC with *EGFR* 19del or 21exon L858R mutations have a lower clinical response rate to ICB combined with chemotherapy. NSCLC patients with *EGFR* ex20ins mutation achieved both better PFS and OS than NSCLC patients with no targetable oncogenes after ICB and chemotherapy treatment (Remon et al., 2020). Therefore, chemotherapy in combination with nivolumab was administered to our patient, resulting in a significant reduction of pleural effusion and relieved dyspnea for more than 5 months. Likewise, in a retrospective single-center case series of patients with NSCLC and *EGFR* ex20ins mutation, two patients received platinum-doublet chemotherapy in combination with pembrolizumab, and both had stable disease (Shah et al., 2021). Thus, according to these reports and our case, ICB combined with chemotherapy can be considered an effective therapeutic strategy in NSCLC patients with *EGFR* ex20 p. N771delinsGF mutation. However, there is a lack of clinical trials to validate this hypothesis, and future clinical trials could be conducted to clarify the efficacy of ICB in combination with chemotherapy in NSCLC patients harboring *EGFR* ex20ins mutation.

In conclusion, our case suggests that phenotypic transition between LUSC and LUAD may occur in lung cancer during treatment. When lung cancer progresses, rebiopsy is helpful in accurately understanding and treating the disease. Moreover, NSCLC with an *EGFR* ex20 p. N771delinsGF mutation can benefit from nivolumab plus docetaxel and carboplatin. ICB plus chemotherapy may be a favorable strategy for NSCLC with *EGFR* ex20 mutation.

## Data Availability

The original contributions presented in the study are included in the article/Supplementary Material, further inquiries can be directed to the corresponding author.
